# Optimization of PTH/PTHrP Hybrid Peptides to Derive a Long‐Acting PTH Analog (LA‐PTH)

**DOI:** 10.1002/jbm4.10367

**Published:** 2020-05-30

**Authors:** Hiroshi Noda, Makoto Okazaki, Eri Joyashiki, Tatsuya Tamura, Yoshiki Kawabe, Ashok Khatri, Harald Jueppner, John T Potts, Thomas J Gardella, Masaru Shimizu

**Affiliations:** ^1^ Research Division Chugai Pharmaceutical Co., Ltd Shizuoka Japan; ^2^ Endocrine Unit Massachusetts General Hospital Boston MA USA

**Keywords:** PARATHYROID‐RELATED DISORDERS, DISORDERS OF CALCIUM/PHOSPHATE METABOLISM, HORMONE REPLACEMENT/RECEPTOR MODULATORS, THERAPEUTICS, PRECLINICAL STUDIES, ANIMAL MODELS, PTH/ViT D/FGF23, CELL/TISSUE SIGNALING, ENDOCRINE PATHWAYS

## Abstract

Prolonged signaling at the parathyroid hormone receptor 1 (PTHR1) correlates with the capacity of a ligand to bind to a G protein‐independent receptor conformation (R^0^). As long‐acting PTH (LA‐PTH) ligands hold interest as potential treatments for hypoparathyroidism (HP), we explored the structural basis in the ligand for stable R^0^ binding and prolonged cAMP signaling. A series of PTH/PTHrP hybrid analogs were synthesized and tested for actions in vitro and in vivo. Of the series, [Ala^1,3,12^,Gln^10^,Arg^11^,Trp^14^]‐PTH(1‐14)/PTHrP(15–36) (M‐PTH/PTHrP) bound with high affinity to R^0^, induced prolonged cAMP responses in UMR106 rat osteoblast‐derived cells, and induced the most prolonged increases in serum calcium (sCa) in normal rats. Daily s.c. injection of M‐PTH/PTHrP into thyroparathyroidectomized (TPTX) rats, a model of HP, normalized sCa without raising urine Ca. In contrast, oral alfacalcidol, a widely used treatment for HP, normalized sCa, but induced frank hypercalciuria. M‐PTH/PTHrP exhibited low solubility in aqueous solutions of neutral pH; however, replacement of Leu18, Phe22, and His26 with the less hydrophobic residues, Ala, Ala, and Lys, at those respective positions markedly improved solubility while maintaining bioactivity. Indeed, we recently showed that the resultant analog [Ala^18,22^,Lys^26^]‐M‐PTH/PTHrP or LA‐PTH, effectively normalizes sCa in TPTX rats and mediates prolonged actions in monkeys. These studies provide useful information for optimizing PTH and PTHrP ligand analogs for therapeutic development. © 2020 The Authors. *JBMR Plus* published by Wiley Periodicals, Inc. on behalf of American Society for Bone and Mineral Research.

## Introduction

Hypoparathyroidism (HP) is a rare disorder of calcium (Ca) and phosphate metabolism that most often arises as a result of parathyroid gland damage or removal during surgery of the thyroid gland, but is also associated with hereditary or acquired abnormalities, such as certain autoimmunity disorders, DiGeorge syndrome, mitochondrial dysfunction, and activating mutations of the Ca‐sensing receptor.^1^, ^2^, ^3^, ^4^ Traditional therapy for HP is a regimen of oral vitamin D (typically, 1,25(OH)_2_‐vitamin D3) and Ca supplementation, but this treatment, although often effective, can be associated with marked variations in blood Ca, hypercalciuria, renal deterioration, and even nephrocalcinosis.^5^, ^6^ Recent clinical investigations on PTH‐based modes of hormone‐replacement therapy, including a double‐blind, placebo‐controlled randomized phase 3 study in which PTH(1–84) was given daily by s.c. injection,^7^ have demonstrated sufficient effectiveness, such that PTH(1–84) has been approved by the US Food and Drug Administration as a treatment option. Although this represents an important advance in the treatment of the disease, challenges persist, particularly in the capacity to maintain a steady‐state level of blood Ca without excessive urinary Ca (uCa) excretion.

We have previously reported that [Ala^1,3,12,18,22^,Gln^10^,Arg^11^,Trp^14^,Lys^26^]‐PTH(1‐14)/PTHrP(15–36) is a long‐acting PTH analog, and hence called LA‐PTH, which produces sustained calcemic responses in thyroparathyroidectomized (TPTX) rats and in normal monkeys.^8^ Not reported are the extensive structure–activity relationship studies that led to the development of this analog, as well as the evidence that LA‐PTH is the most desirable ligand analog for the intended purpose. In this work, we characterize the series of PTH/PTHrP hybrid molecules that formed the basis for LA‐PTH development. Our design used the carboxyl terminal portion of PTHrP based on the evidence that it bound with higher affinity to the extracellular domain (ECD) of the PTH receptor, as compared with the PTH C‐terminal segment,^9^ and we further included the optimized N‐terminal portion of PTH modified with the activity‐enhancing “M” substitutions (Ala^1,3,12^,Gln^10^,Arg^11^,Trp^14^) that we identified in previous work on the PTH(1‐14) scaffold.^10^, ^11^, ^12^ We synthesized and tested seven different hybrid molecules with various boundaries between the N‐terminal M–PTH and C‐terminal PTHrP segments ranging from positions 11/12 to positions 30/31. We tested these different hybrid compounds for PTHR1 binding affinity in vitro, assessing both the G protein‐coupled and uncoupled receptor conformations, RG and R^0^, respectively.^13^ Our previous studies revealed that relative affinity for the R^0^ conformation correlates with duration of cAMP signaling.^14^, ^15^ Most importantly, we evaluated the analogs in vivo and thus assessed their capacities to stimulate a calcemic response in normal rats after a single i.v. administration. These evaluations identified the most potent PTH/PTHrP hybrid analog as M‐PTH(1–14)/PTHrP(15–36). However, in our initial assessment of this analog, we found it to have relatively low solubility in aqueous solutions at neutral pH. With further investigation, we identified several amino acid substitutions in the C‐terminal PTHrP region that led to improved solubility while retaining high R^0^ binding affinity, as well as prolonged in vivo calcemic actions. It was this work that led to the choice of [Ala^1,3,12,18,22^,Gln^10^,Arg^11^,Trp^14^,Lys^26^]‐PTH(1‐14)/PTHrP(15–36) as an optimized long‐acting PTH analog (LA‐PTH). Here we present key findings that led to the selection of LA‐PTH as the optimum ligand in the series, and provide further data that compare the Ca‐mobilizing actions of this type of hybrid PTH/PTHrP analog with those of a vitamin D analog in TXPTX rats.

### Materials and Methods

#### Peptides

All peptides used were based on the human PTH or PTHrP amino acid peptide sequence, except for the PTH(1–34) analog used for radiolabeling, which was based on the rat PTH sequence. Specific analogs assessed for function were PTH(1–34), LA‐PTH ([Ala^1,3,12^,Gln^10^,Arg^11^,Trp^14^]PTH(1‐14)/[Ala^18,22^,Lys^26^]PTHrP(15‐36)COOH), and M‐PTH(1‐15) ([Ala^1,12^,Aib^3^,Nle^8^,Gln^10^,Har^11^,Trp^14^,Tyr^15^]‐PTH(1‐15)NH_2_)—in which Aib is α‐aminoisobutyric acid, Har is homoarginine, and Nle is norleucine. The radioligands used were ^125^I‐PTH(1–34), (^125^I‐[Nle^8,21^,Tyr^34^]‐rat PTH(1‐34)NH_2_), and ^125^I‐M‐PTH(1‐15). They were prepared by chloramine‐T‐based radioiodination, followed by reversed‐phase HPLC purification. Peptides were prepared by conventional chemical synthesis. Peptide identity was established by mass spectrometry; purity was confirmed to be greater than 95% by reverse‐phase HPLC.

#### In vitro characterization

##### Cell culture and transfection

GP‐2.3 cells, an HEK‐293 cell‐ (ATCC CRL‐1573) derived cell line that stably expresses the luciferase‐based pGloSensor‐22F (GloSensor) cAMP reporter plasmid (Promega Corp., San Luis Obispo, CA, USA) along with the human PTHR1, were cultured in DMEM (Thermo Fisher Scientific, Waltham, MA, USA) supplemented with FBS (10%). Cells were seeded into white 96‐well plates for GloSensor cAMP assays, and used for assay 2 to 3 days after the monolayer became confluent.^16^ UGS‐56 cells were derived from the UMR106 rat osteoblastic cell line by stably transfecting the cells to express the GloSensor‐cAMP reporter. B64 cells are a derivative of the porcine kidney cell line, LLC‐PK1 that stably expresses the human PTHR1. COS‐7 cells (ATCC CRL‐1661) were seeded into 10‐cm dishes and transiently transfected to express the human PTHR1 with or without a high‐affinity, negative‐dominant Gαs subunit; 2 days after transfection, the cells were harvested for membrane preparations.^13^ All transfections were performed using the Fugene HD reagent (Promega Corp.).

##### cAMP signaling assays

Changes in cAMP levels were assessed in intact GP‐2.3 or UGS‐56 cells via the (GloSensor) cAMP reporter. The intact cells in white 96‐well plates were preloaded with luciferin in CO_2_‐independent culture media (Thermo Fisher) for 20 min at room temperature, then treated with a test ligand diluted in the same media and placed into a Envision plate reader (PerkinElmer, Waltham, MA, USA) for an additional 30 min, during which time cAMP‐dependent luminescence was measured at 2‐min intervals (ligand‐on phase). Ligand dose–response curves were generated by plotting ligand concentrations versus the area‐under‐the‐curve (AUC) of the time versus luminescence plot obtained at each ligand dose and expressing that AUC as a percent of the maximum AUC observed in the same experiment for PTH(1–34). For washout responses, the cells were treated as above, and after the ligand‐on phase, the plate was removed from the plate reader, the cells were rinsed with media, then fresh media containing only luciferin was added and luminescence was measured for an additional 90 min (washout phase). EC_50_ values were determined using the 4‐parameter sigmoidal dose–response equation in Prism 8.0 (GraphPad Software, Inc., La Jolla, CA, USA).

##### Competition binding assays

Ligand binding to the human PTHR1 in R^0^ and RG conformations were assessed by competition methods using membranes prepared from COS‐7 cells and either ^125^I‐PTH(1–34) (R^0^) or ^125^I‐M‐PTH(1‐15) (RG) as tracer radioligand and GTPγS (1 × 10^−5^ M) included in the R^0^ assays.^13^ Binding reactions were performed in 96‐well vacuum filtration plates and were incubated at room temperature for 90 min; the plates were then processed by vacuum filtration; after washing, the filters were removed and counted for gamma irradiation. Nonspecific binding was determined in reactions containing an excess (5 × 10^−7^ M) of unlabeled PTH(1–34). Curves were fit to the data using a 4‐parameter sigmoidal dose–response equation.

##### Peptide solubility

Peptides were initially dissolved in 10 mM acetic acid to obtain a stock solution of peptide at 1.0 × 10^−3^ to 2.0 × 10^−3^ M. For assay, a stock solution was diluted in aqueous vehicle (pH 5.0) or assay buffer (pH 7.4) to concentrations of 1.0 – 3.0 × 10^−5^ M, at which all peptides exhibited good solubility. For certain peptides, including, the M‐PTH/PTHrP hybrid and PTHrP(1–36), precipitation or turbidity was observed in aqueous neutral pH buffer at a higher peptide concentration (>1 × 10^−4^ M), which led us to perform further optimization of the C‐terminal PTHrP portion of the peptide scaffold. To assess solubility, a volume of each stock peptide solution that contained 75‐μg peptide mass was placed, in duplicate, into a 1.5‐cc microcentrifuge tube and lyophilized to dryness. For each peptide, one of the duplicate lyophilized samples was reconstituted in 50 μL of 10‐mM acetic acid to a final concentration of 1.5 mg/cc; the other duplicate sample was reconstituted in 50 μL of PBS, pH 7.4. The solutions were then incubated for 24 hours at room temperature, then centrifuged at 16,000*g* for 2 min at room temperature. A 10‐μL aliquot of the upper supernatant phase of each sample was removed in duplicate and assayed for protein concentration using the BCA protein assay (Pierce, Rockford, IL, USA). The protein concentration (mg/cc) of each sample was then derived from a standard curve generated with bovine serum albumin. The relative solubility in PBS was calculated as: 100 × (protein concentration of PBS supernatant/protein concentration of acetic acid supernatant).

#### In vivo pharmacology

##### Calcemic actions in normal rats

Peptides were diluted in phosphate–citrate‐buffered saline vehicle (prepared by adding 23 mmol/L citric acid/100 mmol/L NaCl added to 25 mmol/L sodium‐phosphate/ 100 mmol/L NaCl until the pH of 5.0 was attained) containing 0.05% Tween‐80 (Tokyo Chemical Industry Co., Ltd., Tokyo, Japan). Six week‐old normal rats were randomly assigned to test groups, and M‐modified PTH/PTHrP hybrid analogs were administrated by a single i.v. administration. Blood was collected in a glass capillary at 2‐min intervals; blood ionized Ca^2+^ was measured by Ca analyzer (GE Healthcare, Piscataway, NJ, USA).

##### Pharmacological studies in TPTX rats

Surgical TPTX was performed on 6‐week old rats (Charles River Laboratories Japan, Inc, Kanagawa, Japan); postsurgical rats exhibiting serum calcium (sCa) levels less than 8.0 mg/dL were selected for subsequent peptide injection studies. TPTX rats were injected i.v. or s.c. with vehicle or vehicle containing a PTH peptide, or orally treated once daily with vehicle (medium‐chain triglyceride; Chugai Pharma Manufacturing, Tokyo, Japan) or vehicle containing alfacalcidol (Chugai Pharma Manufacturing) for 11 days. Blood samples were obtained from the tail or jugular vein immediately before and at times after injection. Urine samples at 0 to 8 hours or 8 to 24 hours, were collected in metabolic cages. sCa and uCa, serum inorganic phosphorus (sPi) and urinary phosphorus (uPi), and creatinine (uCre) were measured with an automatic analyzer (7180; Hitachi, Ltd, Chiyoda, Tokyo, Japan). Urinary deoxypyridinoline (uDpD) was measured using a Metra DPD EIA kit (DS Pharma Biomedical, Osaka, Japan), and the data were normalized to the uCre concentrations. Serum FGF23 was measured using FGF23 ELISA assay (Immunodiagnostik AG, Bensheim, Germany). Serum 1,25‐dihydroxyvitamin D [1,25(OH)_2_D_3_] levels were determined by a ELISA immunoassay (Immunodiagnostic System Ltd, Bolden, UK).

For pharmacokinetic studies, plasma samples were prepared in the presence of protease inhibitors (aprotinin, leupeptin, EDTA). Plasma levels of PTH(1–34) were assessed by ELISA assay (Human PTH(1–34) Specific ELISA Kit; Immutopics Inc, San Clemente, CA, USA), and plasma levels of M‐PTH/PTHrP were assessed by ELISA assay (PTH‐RP 1–34 Human Enzyme Immunoassay Kit; Peninsula Laboratories International, San Carlos CA, USA) using M‐PTH/PTHrP peptides as standard solutions.

##### Monkeys

Male cynomolgus monkeys at age 3 to 4 years were purchased from Hamri Co. Ltd (Tokyo, Japan). Cynomolgus monkeys were randomly assigned and injected s.c. with either vehicle, M‐PTH/PTHrP, or LA‐PTH. Blood samples were collected from the saphenous vein and assessed for sCa or sPi levels.

All animal studies were approved by the Institutional Animal Care and Use Committee of Chugai Pharmaceutical, and were conducted in accordance with the approved protocols and the Guidelines for the Care and Use of Laboratory Animals at Chugai. Chugai Pharmaceutical is fully accredited by the Association for Assessment and Accreditation of Laboratory Animal Care (AAALAC) International.

### Statistical analysis

Data are represented as the mean ± SE and statistical significance was determined using JMP Ver.11 (SAS Institute Japan, Tokyo, Japan). A Dunnet test was performed to assess the significant differences in the PTH(1–34), M‐PTH/PTHrP, or LA‐PTH groups compared with the vehicle groups. A Student's *t* test was used to assess the significance between two data sets (TPTX‐vehicle versus sham).

## Results

### Binding and cAMP signaling properties of PTH/PTHrP hybrid peptides in vitro and calcemic actions in normal rats

**Figure 1:**
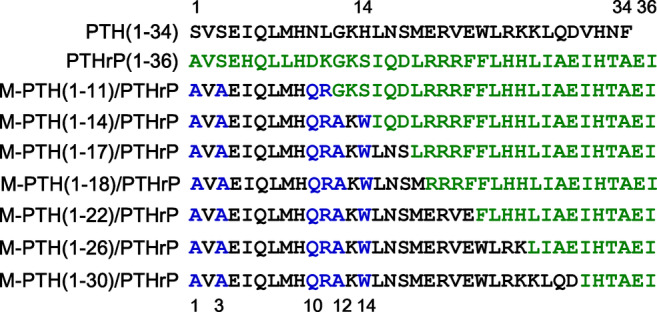
Primary structure of PTH, PTHrP, and M‐modified PTH/PTHrP hybrid analogs. Amino acid sequences of human PTH(1–34), human PTHrP(1–36), and PTH/PTHrP hybrid analogs. PTH and PTHrP residues are colored black and green, respectively, and the N‐terminal “M” modifications at positions 1, 3, 10, 11, 12, and 14 in PTH are colored blue. M‐PTH(1‐14)/PTHrP(15–36) analog is also referred to as M‐PTH/PTHrP in the text.

The primary structures of the M‐PTH/PTHrP hybrid peptides evaluated in this study are shown in Figure 1, and their PTHR1‐binding properties, assessed in membranes prepared from COS‐7 cells transfected to express the human PTHR1, as well as their cAMP signaling properties, assessed in intact COS‐7 cells transfected as above, are reported in Tables 1 and 2. Affinities at the RG PTHR1 conformation were similar for all analogs tested, whereas each hybrid peptide bound to the R^0^ conformation with an affinity several‐fold higher than that of PTH(1–34), and only slight differences in affinity were detected between any of the hybrid analogs (Tables 1 and 2). All M‐PTH/PTHrP hybrid analogs exhibited more prolonged cAMP signaling responses on human PTHR1, as compared with PTH(1–34) and PTHrP(1–36) (Fig. S1).

**Table 1:** Binding to the RG and R^0^ Conformations of Human PTHR1^a^

	IC_50_ (nM)							
	R^0^				RG			
		*n*	*p* versus			*n*	*p* versus	
			PTH(1‐34)	PTHrP(1‐36)			PTH(1‐34)	PTHrP(1‐36)
PTH(1‐34)	10.4 ± 3.7	8	1.00	0.67	0.16 ± 0.08	8	1.00	0.30
PTHrP(1‐36)	8.20 ± 3.41	3	0.67	1.00	0.07 ± 0.01	3	0.30	0.30
M‐PTH(1‐11)/PTHrP(12‐36)	2.14 ± 0.47	3	0.06	0.22	0.55 ± 0.15	2	0.17	0.19
M‐PTH(1‐14)/PTHrP(15‐36)	2.69 ± 0.99	3	0.08	0.24	0.67 ± 0.05	3	0.0006	0.0064
M‐PTH(1‐17)/PTHrP(18‐36)	1.91 ± 0.39	4	0.06	0.20	0.23 ± 0.02	4	0.42	0.00
M‐PTH(1‐18)/PTHrP(19‐36)	1.66 ± 0.22	4	0.05	0.19	0.13 ± 0.02	4	0.72	0.02
M‐PTH(1‐22)/PTHrP(23‐36)	7.80 ± 2.55	3	0.58	0.93	0.67 ± 0.02	3	0.0004	0.0001
M‐PTH(1‐26)/PTHrP(27‐36)	1.73 ± 0.75	5	0.05	0.19	0.13 ± 0.04	5	0.73	0.21
M‐PTH(1‐30)/PTHrP(31‐36)	2.98 ± 0.48	4	0.09	0.26	0.21 ± 0.03	4	0.60	0.013

Equilibrium competition binding reactions were performed either in the presence (R0) or absence (RG) of GTPgammaS, and RG reactions used membranes from cells transfected with a high‐affinity GαS‐dominant‐negative mutant in addition to the PTHR1. Data are means ± SEM of the number of experiments indicated by *n*; *p* = Student's *t* test versus PTH(1‐34) or PTHrP(1‐36).

a IC_50_ values are peptide concentrations (nM) that resulted in 50% inhibition of binding of ^125^I‐labeled PTH(1‐34) radioligand (R0) or ^125^I‐labeled M‐PTH(1‐15) radioligand (RG) to the hPTHR1 expressed in membranes of transiently transfected COS‐7 cells.

**Table 2:** cAMP Responses of Human PTHR1 in GP‐2.3 Cells^a^

	EC_50_ (nM)			E_Max_ (%)		
		*p* versus			*p* versus	
		PTH(1‐34)	PTHrP(1‐36)		PTH(1‐34)	PTHrP(1‐36)
PTH(1‐34)	1.4 ± 0.6	1.00	0.55	100 ± 0	1.00	0.81
PTHrP(1‐36)	1.82 ± 0.45	0.55	1.00	99 ± 3	0.81	1.00
M‐PTH(1‐11)/PTHrP(12‐36)	4.46 ± 1.27	0.088	0.13	103 ± 3	0.46	0.46
M‐PTH(1‐14)/PTHrP(15‐36)	5.18 ± 1.07	0.029	0.044	105 ± 4	0.37	0.36
M‐PTH(1‐17)/PTHrP(18‐36)	6.78 ± 1.98	0.067	0.084	105 ± 5	0.36	0.34
M‐PTH(1‐18)/PTHrP(19‐36)	6.01 ± 1.59	0.055	0.073	107 ± 5	0.28	0.26
M‐PTH(1‐22)/PTHrP(23‐36)	2.58 ± 0.26	0.12	0.20	104 ± 4	0.39	0.37
M‐PTH(1‐26)/PTHrP(27‐36)	1.79 ± 0.41	0.56	0.97	106 ± 5	0.38	0.35
M‐PTH(1‐30)/PTHrP(31‐36)	1.52 ± 0.14	0.81	0.56	106 ± 4	0.21	0.21

EC50 values are peptide concentrations that stimulated 50% of the maximum response (EMax) for that peptide. Data were derived from the area‐under‐the‐curves (AUC) of time‐course luminescence responses measured at multiple doses and calculated as a % of the maximum AUC response attained in each assay with PTH(1‐34), the average of which was 104 ± 19 × 10^5^ counts per second (cps) • min, and the corresponding basal value (not subtracted) was 0.52 ± 0.13 × 10 ^ cps • min. Data are means ± SEM of four experiments; *p* values determined by Student's *t* test compare responses to PTH(1‐34) and PTHrP(1‐36).

a cAMP responses were measured in GP‐2.3 cells (HEK293 cells stably transfected to express the hPTHR1 and GloSensor cAMP reporter).

**Figure 2:**
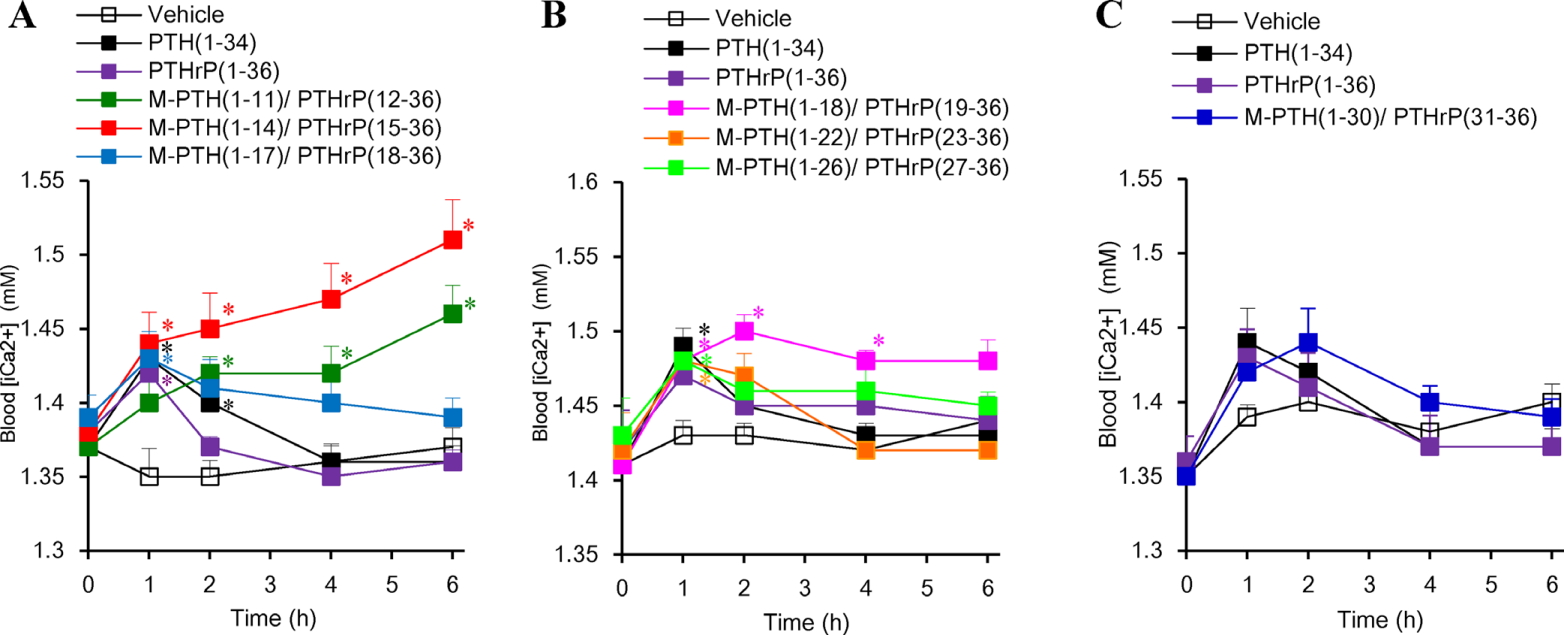
Calcemic actions of M‐PTH/PTHrP hybrid analogs in normal rats. M‐PTH/PTHrP hybrid analogs, PTH(1–34) and PTHrP(1–36) at 5.0 nmol/kg were i.v. administered, and blood ionized calcium (Ca^2+^), collected from the tail vein, was measured at the indicated time points. (*A*) M‐PTH(1‐11)/PTHrP(12–36) to M‐PTH(1‐17)/PTHrP(18–36) hybrid analogs in experiment 1. (*B*) M‐PTH(1‐18)/PTHrP(19–36) to M‐PTH(1–26)/PTHrP(27–36) in experiment 2. (*C*) M‐PTH(1–30)/PTHrP(31–36) in experiment 3. Data are means ± SEM; *n* = 6. *p < 0.05 versus vehicle.

Having established adequate receptor‐binding properties in vitro, we next tested the calcemic actions of the M‐modified PTH/PTHrP hybrid analogs in normal rats. The peptides were administered by a single i.v. injection at a dose of 5.0 nmol/kg of body weight for each analog (Fig. 2 *A*–*C*), as well as at multiple doses (Fig. S2 *A*–*C*). M‐PTH(1‐11)/PTHrP(12–36) and M‐PTH(1‐14)/PTHrP(15–36) at 5 nmol/kg significantly increased blood Ca^2+^ levels at 1 hour, and further increased blood Ca^2+^ levels up to 6 hours after administration (Fig. 2 *A*). M‐PTH(1‐18)/PTHrP(19–36) increased blood Ca^2+^ at 1 hour, and sustained levels for up to 6 hours. M‐PTH(1‐17)/PTHrP(18–36), M‐PTH(1–22)/PTHrP(23–36), M‐PTH(1–26)/PTHrP(27–36), and M‐PTH(1–30)/PTHrP(31–36) increased blood Ca^2+^ levels more transiently, with peak responses occurring at 1 to 2 hours, and then blood Ca^2+^ levels returning to basal levels gradually. As M‐PTH(1‐14)/PTHrP(15–36) exerted the most robust and prolonged calcemic action at the 5 mmol/kg dose, we selected this analog, hereafter referred to as M‐PTH/PTHrP, for further testing.

### In vivo calcemic actions of M‐PTH/PTHrP in thyroparathyroidectomized rats by a single administration

**Figure 3:**
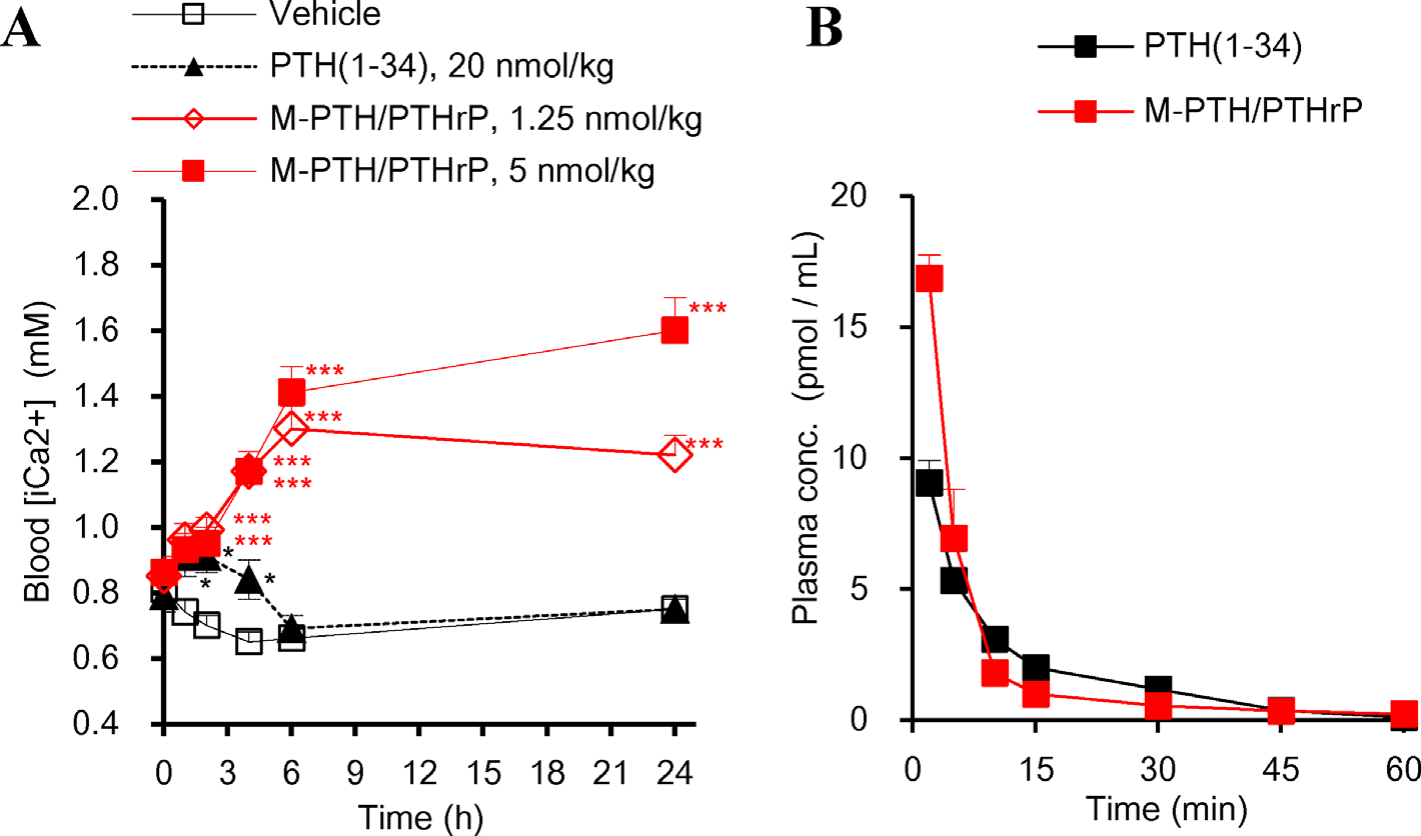
Pharmacodynamic and pharmacokinetic profiles of M‐PTH/PTHrP and PTH(1–34) in rats. (*A*) Effects of M‐PTH/PTHrP and PTH(1–34) on blood ionized Ca in TPTX rats by i.v. injection. M‐PTH/PTHrP and PTH(1–34) were injected intravenously in TPTX rats at the indicated doses, and blood samples were collected from the tail vein measured for ionized Ca; data are means ± SE; *n* = 5 or 3 (PTH(1–34)). *p < 0.05, **p < 0.01, ***p < 0.005 M‐PTH/PTHrP or PTH(1–34) versus TPTX‐vehicle. Blood ionized Ca in TPTX rats injected with M‐PTH/PTHrP at 1.25 and 5.0 nmol/kg at 1 hour were statistically higher than those in vehicle‐injected TPTX rats (*p < 0.05). (*B*) Pharmacokinetics of M‐PTH/PTHrP and PTH(1–34). M‐PTH/PTHrP and PTH(1–34) each at a dose of 10 nmol/kg were i.v. administered, and blood samples were collected from the jugular vein in the presence of proteinase inhibitors (Aprotinin, Leupeptin, and EDTA) and the plasma samples were assessed for peptide concentration using a hPTHrP(1–34)‐targeted EIA for M‐PTH/PTHrP and an hPTH(1–34)‐targeted ELISA for PTH(1–34). Data are means ± SE; *n* = 4 (M‐PTH/PTHrP) or 3 (PTH(1–34)).

We then evaluated the capacity of M‐PTH/PTHrP to modulate blood Ca^2+^ levels in TPTX rats by a single i.v. administration and in comparison with PTH(1–34) (Fig. 3 *A*). These TPTX rats exhibit moderate hypocalcemia in the basal state and thus represent a model of surgical HP. Injection of M‐PTH/PTHrP at each of two doses resulted in a significant increase in blood Ca^2+^ levels. Whereas the higher dose of 5.0 nmol/kg resulted in frank hypercalcemia (Ca^2+^ >1.6 mM) by 24‐hour postinjection, the lower dose of 1.25 nmol/kg restored blood Ca^2+^ levels to nearly the normal level (approximately 1.2 mM) by 6 hours, and the restorative effect persisted for up to 24 hours. At the even higher dose of 20 nmol/kg, PTH(1–34) produced a much smaller and more transient increase in blood Ca^2+^.

We then compared the pharmacokinetic properties of M‐PTH/PTHrP and PTH(1–34) by i.v. injecting the peptides at equivalent doses (10 nmol/kg) into normal rats and measuring the blood concentrations of the peptides at times after injection by ELISA immunoassay (Fig. 3 *B*). Initial experiments established the M‐PTH/PTHrP analog cross‐reacted adequately with an ELISA assay targeted to PTHrP. Upon i.v. injection, each peptide disappeared rapidly from the circulation, such that the plasma concentrations declined to the lowest detection level by the 60‐min time point. This rapid decline in serum ligand concentrations, coupled with a prolonged pharmacodynamic profile of M‐PTH/PTHrP observed in intact and TPTX rats, is consistent with the rapid clearance rate and prolonged calcemic responses observed for LA‐PTH and similar M‐PTH analogs in our previous studies.^8^, ^14^

### Effects of repeated daily administrations of M‐PTH/PTHrP in thyroparathyroidectomized rats

**Figure 4:**
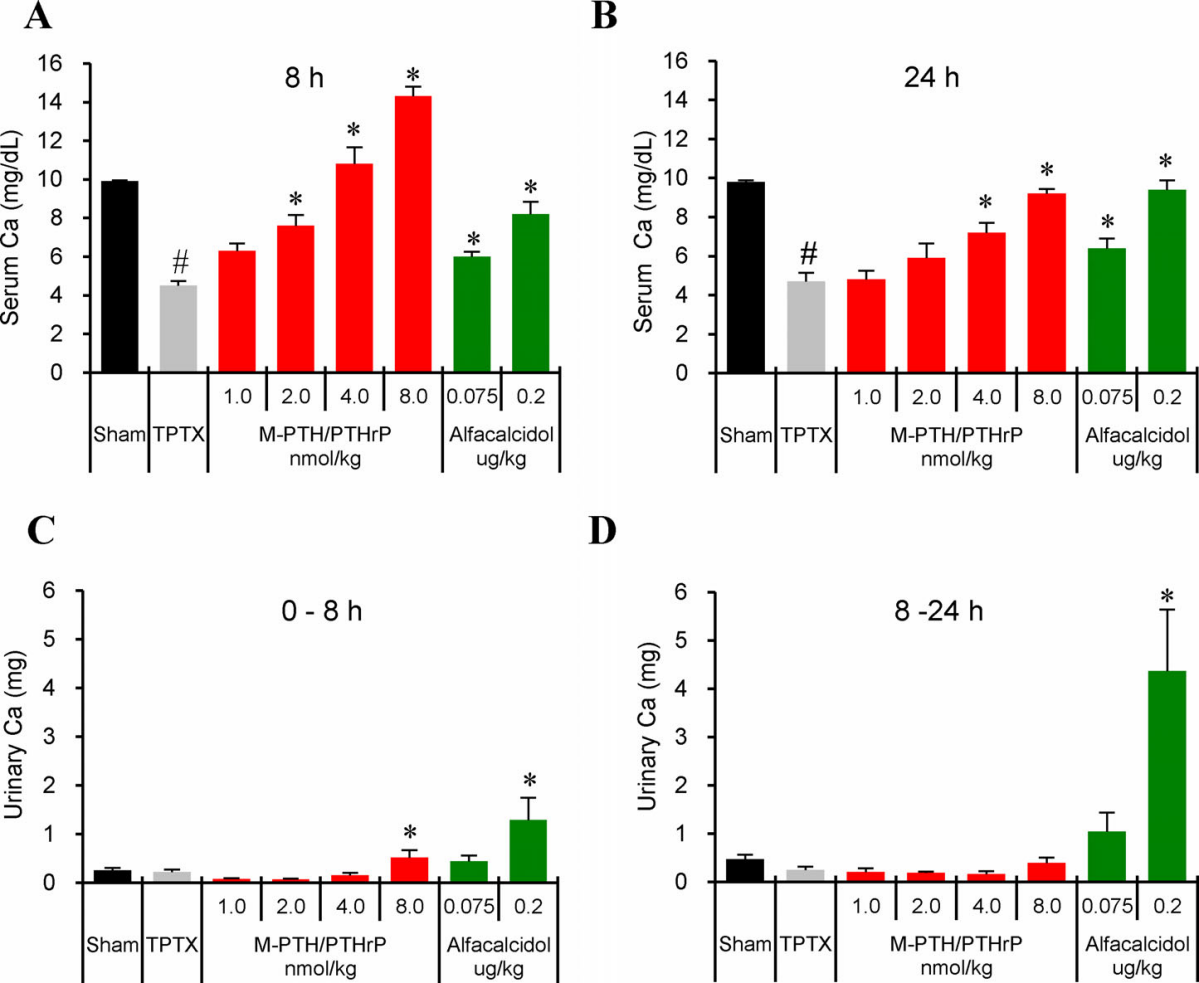
Effects of 11‐day treatment (12‐day study) with M‐PTH/PTHrP or alfacalcidol on serum calcium (sCa) in thyroparathyroidectomized (TPTX) rats. TPTX rats were treated for 11 days with daily s.c. injection of either vehicle or M‐PTH/PTHrP, or with daily oral alfacalcidol at the indicated doses, and sham‐surgery control rats were s.c. injected daily with vehicle. The levels of Ca in serum and urine were assessed. Blood samples were collected from the jugular vein at 8 hours (*A*) and at 24 hours (*B*) after the last treatment administration, and assessed for total sCa. Total urine was collected in metabolic cages over the time intervals of 0 to 8 hours (*C*), and 8 to 24 hours (*D*) and assessed for total urinary Ca. Data are means ± SEM; *n* = 6 or 5 (2.0 and 4.0 nmol/kg of M‐PTH/PTHrP); *p < 0.05 M‐PTH/PTHrP versus TPTX‐vehicle; #*p* < 0.05 TPTX vehicle versus sham.

Next, we studied the effects of M‐PTH/PTHrP on total sCa in comparison with the vitamin D analog, alfacalcidol (1α‐hydroxycholecalciferol), over a course of 12 days in TPTX rats. M‐PTH/PTHrP dose‐dependently increased sCa levels, as measured at 8 and 24 hours after the last administration on day12 (Fig. 4 *A*,*B*). At a dose of 4.0 nmol/kg, M‐PTH/PTHrP resulted in sCa levels that were comparable to those in the sham‐operated control group, whereas uCa levels, measured at the intervals of 0 to 8 hours and 8 to 24 hours, remained low (Fig. 4 *C*,*D*). At the 8.0 nmol/kg dose of M‐PTH/PTHrP, frank hypercalcemia was observed at 8 hours after administration, and uCa at 0 to 8 hours, and uDpD at 0 to 24 hours increased significantly after the last administration (Figs. 4 *C*,*D,* and S3 *B*). Daily administration of alfacalcidol also dose‐dependently increased sCa levels, and the higher dose of 0.2 μg/kg increased sCa to levels comparable to those of the sham group; however, this dose of alfacalcidol was accompanied by high uCa excretion levels at both 0‐ to 8‐ and 8‐ to 24‐hour time intervals. No significant change in serum 1,25(OH)_2_D_3_ was observed among all groups (Fig. S3 *A*,*B*).

Compared with the sham control animals, the TPTX rats exhibited elevated urine levels of sPi, and daily injection with M‐PTH/PTHrP dose dependently decreased these levels, with the 4.0 nmol/kg dose achieving a sPi level that was comparable to that of the sham controls at 8 and 24 hours (Fig. S4 *A*,*B*). This lowering of sPi was accompanied by a significant increase in uPi excretion over the 0‐ to 8‐hour interval (Fig. S4 *C,D*). Alfacalcidol also normalized sPi levels, especially at the 0.2 μg/kg dose, with a significant increase of uPi excretions. M‐PTH/PTHrP at 4.0 and 8.0 nmol/kg and alfacalcidol at 0.2 μg/kg significantly increased serum levels of FGF23 (Fig. S3 *C*).

### Optimizing solubility of the M‐PTH/PTHrP scaffold to derive LA‐PTH

**Figure 5:**
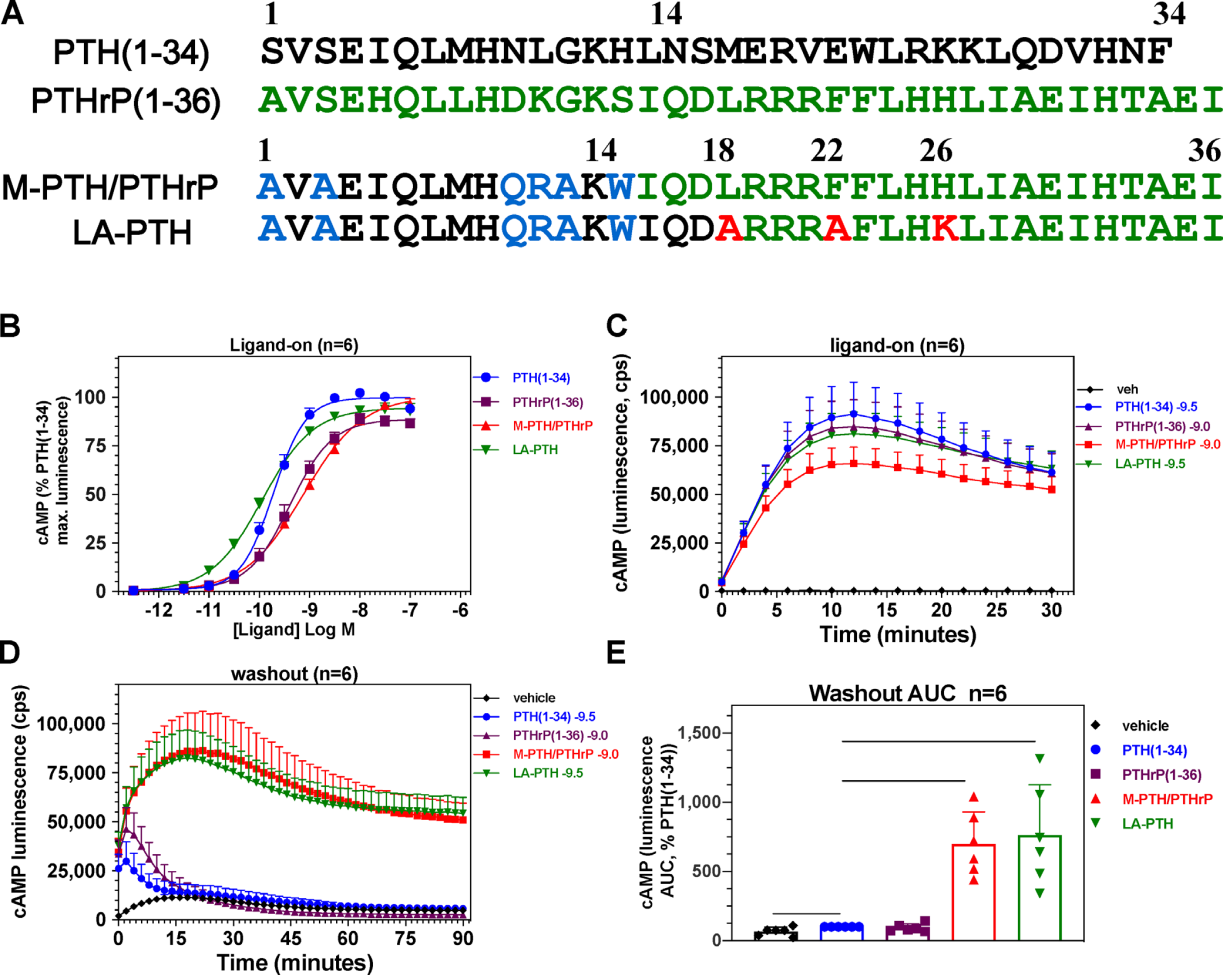
Sequence and functional comparison of M‐PTH/PTHrP and LA‐PTH in a rat osteoblast‐derived cell line. (*A*) Sequence of PTH(1–34), PTHrP(1–36) and hybrid analogs, M‐PTH/PTHrP, and LA‐PTH. (*B*) Dose–response for stimulation of cAMP signaling in a UMR106‐derived rat osteoblastic cell line (UGS‐56 cells). (*C*) Time course of the cAMP‐dependent luminescence response measured at 2‐min intervals in the presence of a near‐EC_50_ concentration of each ligand: PTH (1–34) (0.3 nM), PTHrP (1–36) (1.0 nM), M‐PTH/PTHrP (1 nM), and LA‐PTH (0.3 nM). (*D*) Time course of the cAMP‐dependent luminescence response measured in the same cells as in (*C*), but after washout of unbound ligand. (*E*) Area‐under‐the‐curve (AUC) values of the washout responses shown in (*D*). Data are means (± SEM) of six experiments, each performed in triplicate. Dose–response EC_50_ and washout‐AUC values are reported in Table 3.

In the course of this work, we found that several of the hybrid analogs containing the C‐terminal portion of PTHrP, including M‐PTH/PTHrP, as well as PTHrP(1–36) itself, were poorly soluble at high concentrations (> approximately 0.3 mg/mL) in aqueous buffers at neutral pH. This led us to investigate amino acid substitutions in the C‐terminal PTHrP sequence that could improve peptide solubility yet preserve functional activity. We first assessed for functional effects utilizing the PTHrP(1–28) scaffold peptide, as critical residues of receptor binding are known to be located in the 15–28 domain. An initial alanine scan through the (15–28) segment of PTHrP(1–28) revealed that positions 18, 22, 25, and 26 were tolerant of alanine replacement, for both PTHR1 binding and stimulating cAMP formation (Fig. S5 *A*,*B*). A subsequent “Type” substitution analysis, by which we introduced at each of those four sites, a representative amino acid of each of the main side chain biochemical groups (eg, aromatic, hydrophobic, charged, polar), revealed that each of the four sites, particularly, 18, 22, and 26, were broadly tolerant of substitution. We then explored various combinations of residues at 18, 22, and 26 and identified Ala18, Ala22, and Lys26 as a combination that when introduced into the full‐length scaffold peptide, M‐PTH(1‐14)/PTHrP(15‐36), preserved or even enhanced PTHR1‐binding affinity (Fig. S5 *C*) and cAMP‐stimulating activity, (Fig. 5, Table 3) and improved peptide solubility, (Fig. S5 *E*) and thus resulted in the analog LA‐PTH.

**Table 3:** cAMP Responses in UMR106/GloSensor cells^a^

	EC_50_ (nM)		E_Max_ (%)		Washout AUC (%)	
		*p*		*p*		*p*
PTH(1‐34)	0.20 ± 0.03	1.000	100 ± 0.4	1.00	100 ± 0	1.00
PTHrP(1‐36)	0.46 ± 0.11	0.062	88.4 ± 2.8	0.007	92 ± 11	0.530
M‐PTH/PTHrP	0.72 ± 0.13	0.012	96.2 ± 2.4	0.143	700 ± 85	< 0.0001
LA‐PTH	0.12 ± 0.01	0.020	94.7 ± 2.9	0.105	763 ± 135	0.001

Responses at each ligand concentration were calculated as the area‐under‐the‐curve (AUC) values obtained from the time‐course plots of cAMP‐dependent luminescence versus time measured over 30 min, and then expressed as a percent of the maximum AUC response observed for PTH(1‐34) in each assay, the average of which was 3.13 ± 0.66 × 10−6 counts per second (cps) • min; the corresponding minimum value (not subtracted) was 3.58 ± 2.16 × 10−8 cps • min. Washout values report the AUC of the cAMP‐dependent luminescence versus time measured for each ligand at a near EC50‐concentration over 90 min and expressed as a percent of the response observed for PTH(1‐34) (0.3 nM) in each assay, the average of which was 0.96 ± 0.87 × 10−6 cps • min; and the corresponding value in vehicle‐treated cells (not subtracted) was 0.48 ± 0.1×10−6 cps • min. Data are means ± SEM of six experiments, with duplicate wells in each; *p* = Student's *t* test versus PTH(1‐34).

a cAMP responses were measured in rat osteoblastic UMR‐106/GloSensor (UGS‐56) cells. EC50 values are peptide concentrations (nM) that stimulated 50% of the maximum response (EMax) observed for that peptide.

### Efficacy of M‐PTH/PTHrP and LA‐PTH in thyroparathyroidectomized rats and normal monkeys

**Figure 6:**
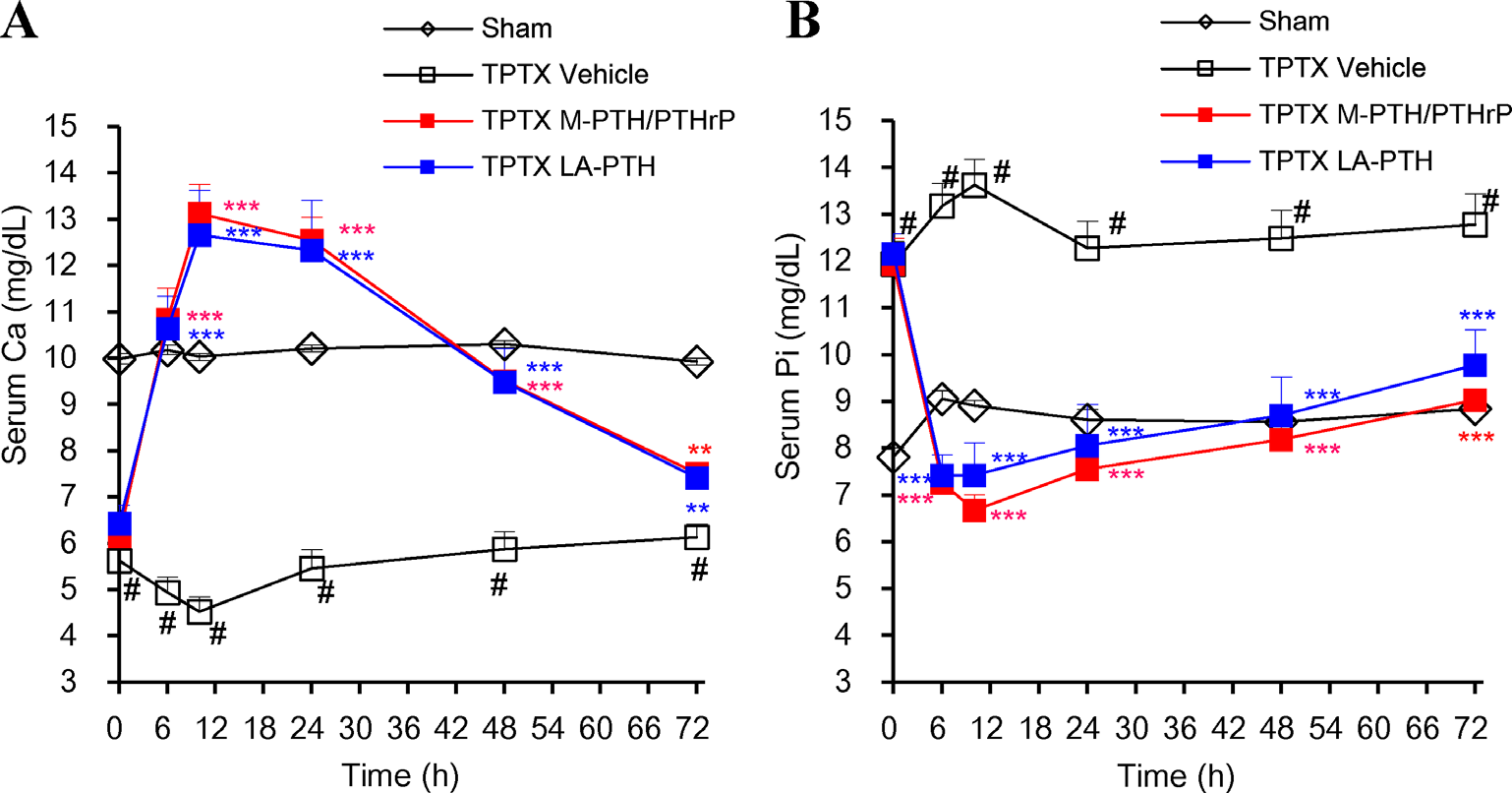
Effects of M‐PTH/PTHrP and LA‐PTH on serum calcium (sCa) and serum phosphorus (sPi) in thyroparathyroidectomized (TPTX) rats. TPTX rats were i.v. injected with vehicle, LA‐PTH, or M‐PTH/PTHrP. Each peptide was at a dose of 1.25 nmol/kg, and sham control rats were injected with vehicle. Blood samples were collected from the jugular vein and assessed for total Ca (*A*) and Pi (*B*). Data are means ± SEM; *n* = 6; *p < 0.05, **p < 0.01, ***p < 0.005 LA‐PTH and M‐PTH/PTHrP versus vehicle.

We then compared the capacity of LA‐PTH to modulate sCa and sPi levels in comparison with M‐PTH/PTHrP by a single i.v. administration in TPTX rats (Fig. 6 *A*,*B*). LA‐PTH and M‐PTH/PTHrP caused comparable increases in total sCa levels over the sham control group that peaked at 10 hours postinjection and persisted at near the sham‐control levels by 48 hours, until returning to near baseline levels by the 72‐hour time point. In parallel, the serum phosphate levels were reduced and persisted near the normal sham‐control levels for at least 72 hours after the dose administration. The efficacy and the duration of the responses induced by LA‐PTH in these TPTX rats were thus comparable to those induced by the M‐PTH/PTHrP parent hybrid peptide.

**Figure 7:**
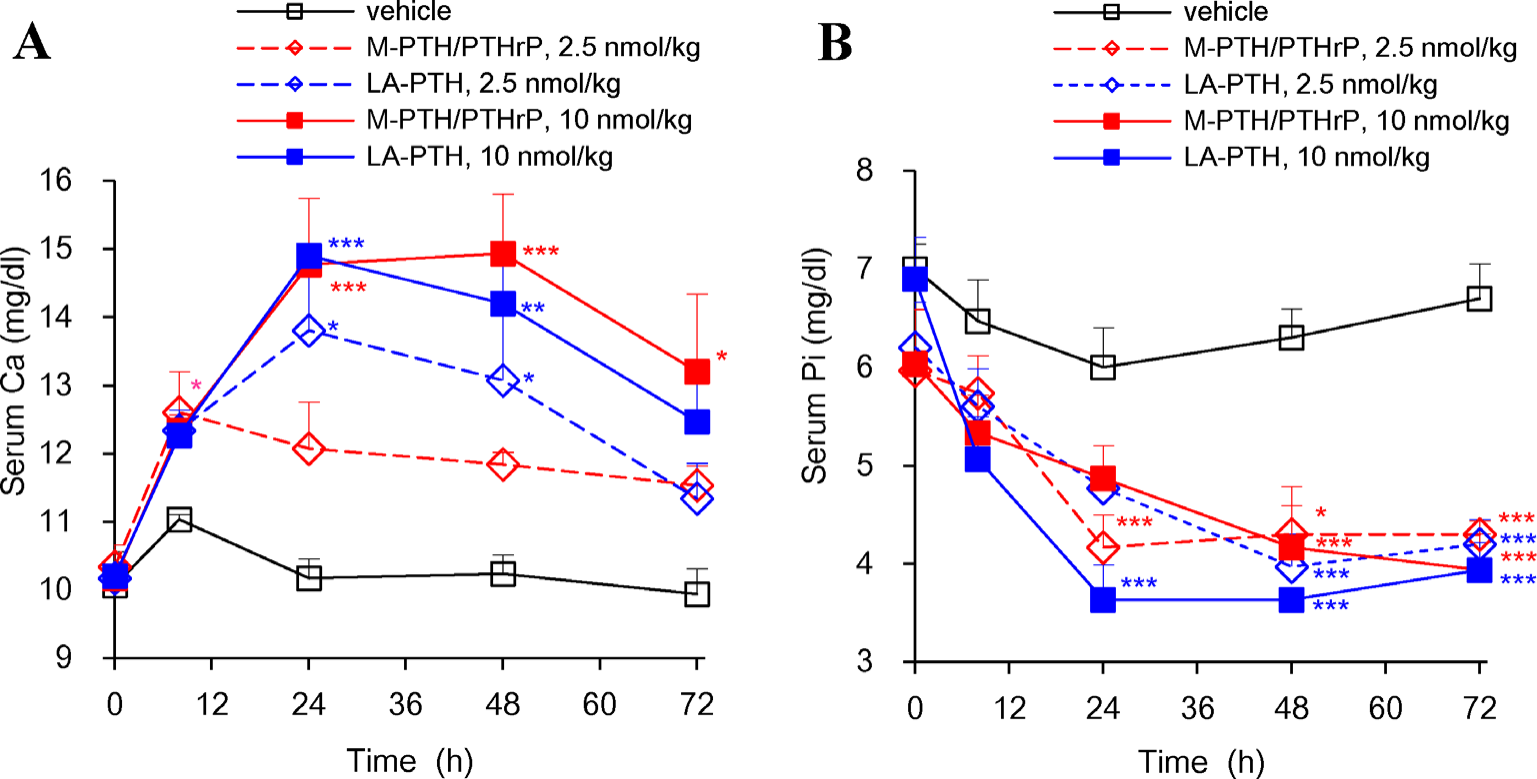
Effects of M‐PTH/PTHrP and LA‐PTH on total serum calcium (sCa) and serum phosphorus (sPi) in normal monkeys. Cynomolgus monkeys were s.c. injected with either vehicle, LA‐PTH, or M‐PTH/PTHrP. Each peptide was at a dose of 2.5 or 10 nmol/kg, and blood samples were collected from the saphenous vein and assessed for total Ca (*A*) and Pi (*B*). Data are means ± SEM; *n* = 3; *p < 0.05, **p < 0.01, ***p < 0.005 LA‐PTH and M‐PTH/PTHrP versus vehicle.

We further compared the effects of LA‐PTH and M‐PTH/PTHrP on total serum sCa and sPi levels in normal monkeys. Upon a single s.c. administration, both LA‐PTH and M‐PTH/PTHrP significantly increased sCa levels by 24 hours after administration, and each analog similarly maintained sCa at higher than 12.0 mg/dL for up to 48 hours (Fig. 7 *A*,*B*). The hypophosphatemic effects of LA‐PTH in monkeys were also comparable to those of M‐PTH/PTHrP as each analog decreased sPi levels for up to 72 hours after administration. These results confirmed that LA‐PTH retains the strong calcemic and hypophosphatemic effects of the parent hybrid peptide, M‐PTH/PTHrP, in TPTX rats as well as in normal monkeys.

## Discussion

In this study, we systematically explored a series of hybrid molecules composed of PTH and PTHrP. The rationale in combining the modified M‐PTH amino terminal region with the carboxyl terminal region of PTHrP to achieve enhanced binding and signaling activity on the PTHR1 was based on the evidence that the PTHrP(15–36) fragment binds with approximately twofold higher affinity to the amino‐terminal ECD portion of the receptor than does the PTH(15–34) fragment,^9^ and that N‐terminal PTH fragments with the “M” modifications, such as M‐PTH(1‐14) and M‐PTH(1‐11), exhibit enhanced binding and signaling interactions with the transmembrane domain region of the PTHR1, as shown by previous structure–activity relationship studies.^10^, ^11^, ^12^ The hybrid analog that proved most active in vivo in Ca‐elevating potency was M‐PTH(1‐14)/PTHrP(15–36). Furthermore, repeated administrations of M‐PTH/PTHrP at an optimal dose (4.0 nmol/kg) normalized sCa levels without increasing uCa, whereas alfacalcidol increased sCa levels, but was associated with hypercalciuria. These results support the concept that the M‐PTH/PTHrP class of ligands could have beneficial pharmacological effects over alfacalcidol in the treatment of hypoparathyroidism, as suggested by a similar comparative study about the effects of a PTHR1 small molecule agonist and alfacalcidol on sCa and uCa has been reported previously.^17^

The low solubility observed for M‐PTH/PTHrP and related analogs containing the C‐terminal PTHrP(15–36) sequence when prepared at high concentrations in aqueous neutral buffer led us to explore amino acid substitutions that would improve solubility yet preserve activity on the PTHR1. Using PTHrP(1–28) as a scaffold peptide for substitution analysis, we thus identified three changes: Leu18➔Ala, Phe22➔Ala, and His26➔Lys, which when combined into the MPTH(1‐14)/PTHrP(15–36) peptide had the desired effect. The resulting analog, [Ala^1,3,12,18,22^,Gln^10^,Arg^11^,Trp^14^,Lys^26^]‐PTH(1‐14)/PTHrP(15–36), termed long‐acting PTH (LA‐PTH), was thus found to be equally potent to the M‐PTH/PTHrP parent hybrid peptide in vitro and in vivo (Figs. 5, 6, 7), and is shown in our previous report to satisfactorily normalize sCa levels in TPTX rats without increases in uCa or causing adverse changes in bone structural parameters, including cortical porosity, when administered daily for 4 weeks at an optimal dose.^8^ The capacity of LA‐PTH and PTH analogs of this class to form highly stable complexes with the PTHR1, which underlies their prolonged pharmacodynamic actions, is highlighted by the recently reported high‐resolution cryo‐electron microscopy structure of LA‐PTH bound to the PTHR1 and a partner heterotrimeric G protein,^18^ as well as the X‐ray crystallographic structure of a PTH(1–34) analog containing N‐terminal modifications similar to those of LA‐PTH bound to a thermo‐stabilized PTHR1 in a G protein‐uncoupled state.^19^ As in each case, the process of structure determination was likely facilitated by the stability provided the ligand analog. It will now be interesting to see, perhaps using computational modeling approaches, how other PTH and PTHrP analogs bind to the PTHR1, and how the binding modes at the PTHR1 may vary, depending on ligand structure. This would extend to the orally available small molecule PTHR1 agonist, PCO371, which has been shown to restore sCa and lower sPi levels in TPTX rats,^17^ and is currently being evaluated in an early‐stage clinical trial for hypoparathyroidism.

In summary, this study shows the ligand optimization path that led to the selection of LA‐PTH as a potent long‐acting PTH analog, modified to make it favorably soluble in aqueous conditions. The study also provides further data on the properties of this class of PTH/PTHrP hybrids to control sCa and sPi without increasing uCa, and illustrates its superiority to treatment with alfacalcidol. The data support further testing of LA‐PTH in hypoparathyroid humans.

## Disclosures

HN, MO, EJ, TT, YK, and MS are employees of Chugai Pharmaceutical Co., Ltd. HJ, TJG, and JTP received a research grant from Chugai Pharmaceutical Co., Ltd.

## Supporting information


**Figure S1** **PTH ligand‐induced cAMP‐signaling responses in GP‐2.3 cells**
Time course of the cAMP‐dependent luminescence response measured at 2‐minute intervals in the presence of each ligand at 10 nM (Ligand on‐phase) and B) Time course of the cAMP‐dependent luminescence response measured at 2‐minute intervals after washout of unbound ligand (Ligand wash‐out phase). GP‐2.3 cells preloaded with luciferin were treated with a concentration of PTH ligand at a concentration of 10 nM for 30 min, and cAMP‐dependent luminescence was measured at two‐minute intervals (ligand‐on phase). The cells were then removed from the plate reader, rinsed twice with media to remove unbound ligand, and then fresh media containing luciferin but lacking ligand was added and luminescence was assessed for additional 90 min (Ligand wash‐out phase). Data are means (± SEM) of four experiments, each performed in triplicate.
**Figure S2**. **In vivo calcemic actions of M‐PTH/PTHrP hybrid analogs in normal rats.**
The indicated M‐PTH/PTHrP hybrid analogs (sequences are shown in Figure 1) along with PTH(1–34) and PTHrP(1–36) controls, were administered at the indicated doses by i.v. injection, and at the indicated time points blood was collected from the tail vein and measured for ionized calcium (Ca^2+^). Panels **A‐C** represent three separate experiments. Data are means ± SEM; n = 6 or 4 (80 nmol/kg of M‐PTH(1‐11)/PTHrP(12–36), M‐PTH(1‐14)/PTHrP(12–36), M‐PTH(1‐17)/PTHrP(18–36) and 1.25 nmol/kg of M‐PTH(1–30)/PTHrP(31–36)). *p < 0.05 versus Vehicle.
**Figure S3. Effects of 11 days treatment (12 days study) of M‐PTH/PTHrP on serum 1,25(OH)**_**2**_ **Vitamin‐D3, urinary deoxypyridinoline (Dpd) and serum FGF23 in TPTX rats.**
Serum and urine samples obtained from the TPTX and sham control rats of the two‐week daily‐administration experiment shown in Figure 4 were analyzed by ELISA for the indicated markers. **A**) serum 1,25(OH)_2_Vitamin‐D3. **B**) urinary deoxypyridinoline (DpD)/Cre. **C**) serum FGF23. Data are means ± SEM; n = 6 or 5 (2.0 and 4.0 nmol/kg of M‐PTH/PTHrP); *p < 0.05 M‐PTH/PTHrP versus TPTX‐vehicle, #*p* < 0.05 TPTX‐vehicle versus sham.
**Figure S4. Effects of 11 days treatment (12 days study) of M‐PTH/PTHrP on serum and urine inorganic phosphorus in TPTX rats.**
Serum and urine samples obtained from the TPTX and sham control rats of the two‐week daily‐administration experiment shown in Figure 4 were analyzed for total inorganic phosphorus (Pi). Data are means ± SEM; n = 6 or 5 (2.0 and 4.0 nmol/kg of M‐PTH/PTHrP); *p < 0.05 M‐PTH/PTHrP versus TPTX‐vehicle, #*p* < 0.05 TPTX‐vehicle versus sham.
**Figure S5. Substitution analysis of the 15–25 region of PTHrP(1–28) and optimization of M‐PTH(1–14)/PTHrP(15–36) solubility.**
**A)** Alanine‐scan analysis of the (15–28) region of PTHrP(1–28) for effects on binding to the RG conformation of the hPTHR1. Native PTHrP(1–28) and analogs thereof containing the indicated single alanine substitutions were assessed at a concentration of 1 nM for the capacity to inhibit binding of ^125^I‐M‐PTH(1‐15) tracer radioligand to membranes prepared from COS‐7 cells transiently transfected to express the hPTHR1 and a high affinity Gγs negative‐dominant mutant. Data are expressed as a percent of the inhibition of specific binding observed with native PTHrP(1–28), which was 39 ± 2 percent of the complete inhibition observed with 3 uM unlabeled M‐PTH(1‐15). **B**) The same peptides were assessed at a concentration of 3 nM for the capacity to stimulate cAMP formation in HKRK‐B64 cells (LLC‐PK1 cells stably expressing the hPTHR1, cAMP measured by RIA). The basal cAMP levels were 1.2 ± 0.1 pmol/well. Data in **A** and **B** are means (±SEM) of three experiments, each performed in triplicate or quadruplicate. **C)** Type‐substitution analysis, in which amino acids with varying side chain structures (e.g., small, bulky, charged, aromatic) are introduced, was performed on residue positions 18, 22, 25 and 26 of PTHrP(1–28), which were identified as tolerant to Ala substitution in the experiments of panels **A** and **B,** and the analogs were assessed at a concentration of 1.0 nM for the capacity to inhibit binding of ^125^I‐M‐PTH(1‐15) to the hPTHR1 (RG) in COS‐7 cell membranes. Data are means (±SEM) of triplicate determinations expressed as a percent of the inhibition of binding observed with native PTHrP(1–28), which was, 41 ± 1 percent of the maximum binding inhibition (NSB, subtracted) observed with unlabeled M‐PTH(1‐15). **D**) The same analogs as in **C** were assessed at a 3.0 nM concentration for the capacity to stimulate cAMP formation in HKRK‐B64 cells. Data are means (±SEM) of three experiments, each performed in duplicate. Basal cAMP levels were 0.3 ± 0.1 pmole/well. **E**) Peptide solubility in PBS was assessed by incubating each indicated peptide at a concentration of 1.5 mg/ml in either PBS at pH 7.4 or in 10 mM acetic acid for 24 h, and then, after centrifugation (15,000xg/ 20 mins.) determining the peptide concentrations of the upper phases and expressing each peptide concentration in the PBS supernatants as a percent of the concentration of the same peptide in the acetic acid supernatant. Data are means (±SEM) of duplicate determinations of a single experiment representative of three others.
