# Supplementary figures and images for: Optimization of PTH/PTHrP Hybrid Peptides to Derive a Long‐Acting PTH Analog (LA‐PTH)
Source: JBMR Plus. 2020 May 30;4(7):e10367. doi: 10.1002/jbm4.10367 (PMC7340446; doi:10.1002/jbm4.10367)

A

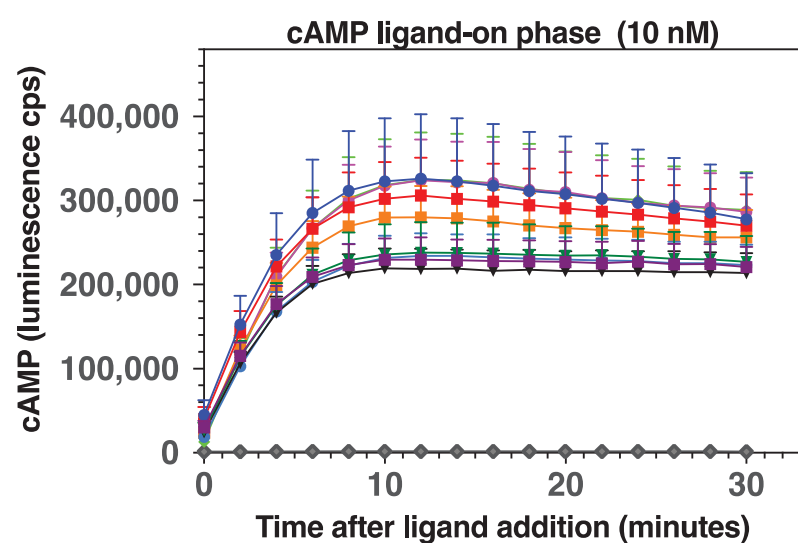

B

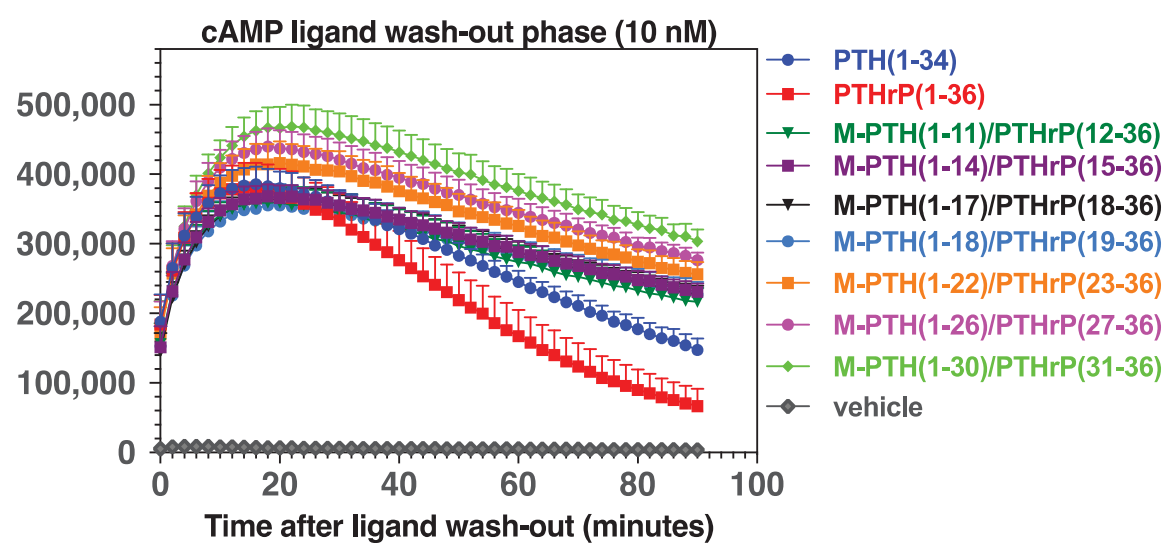

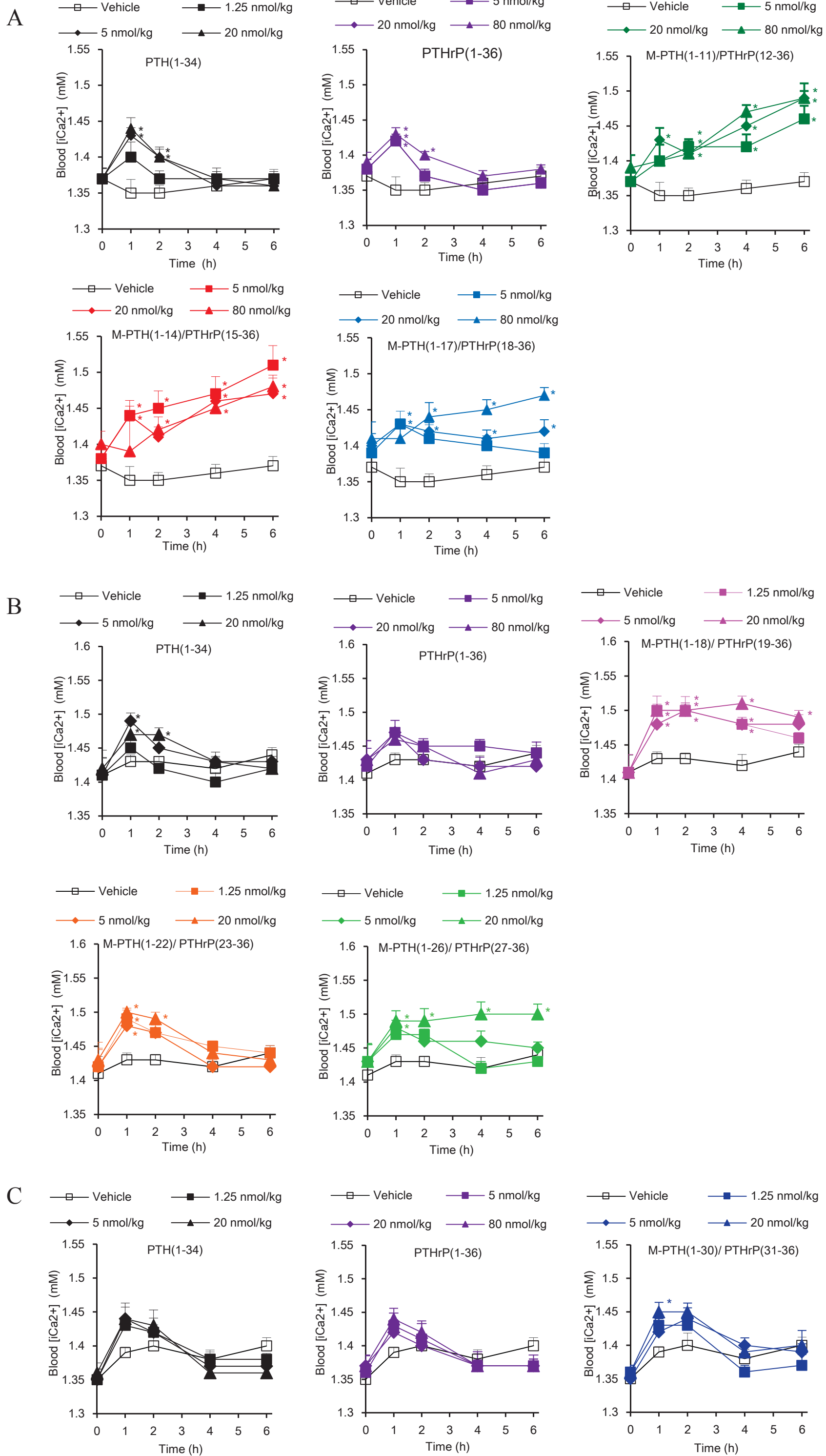

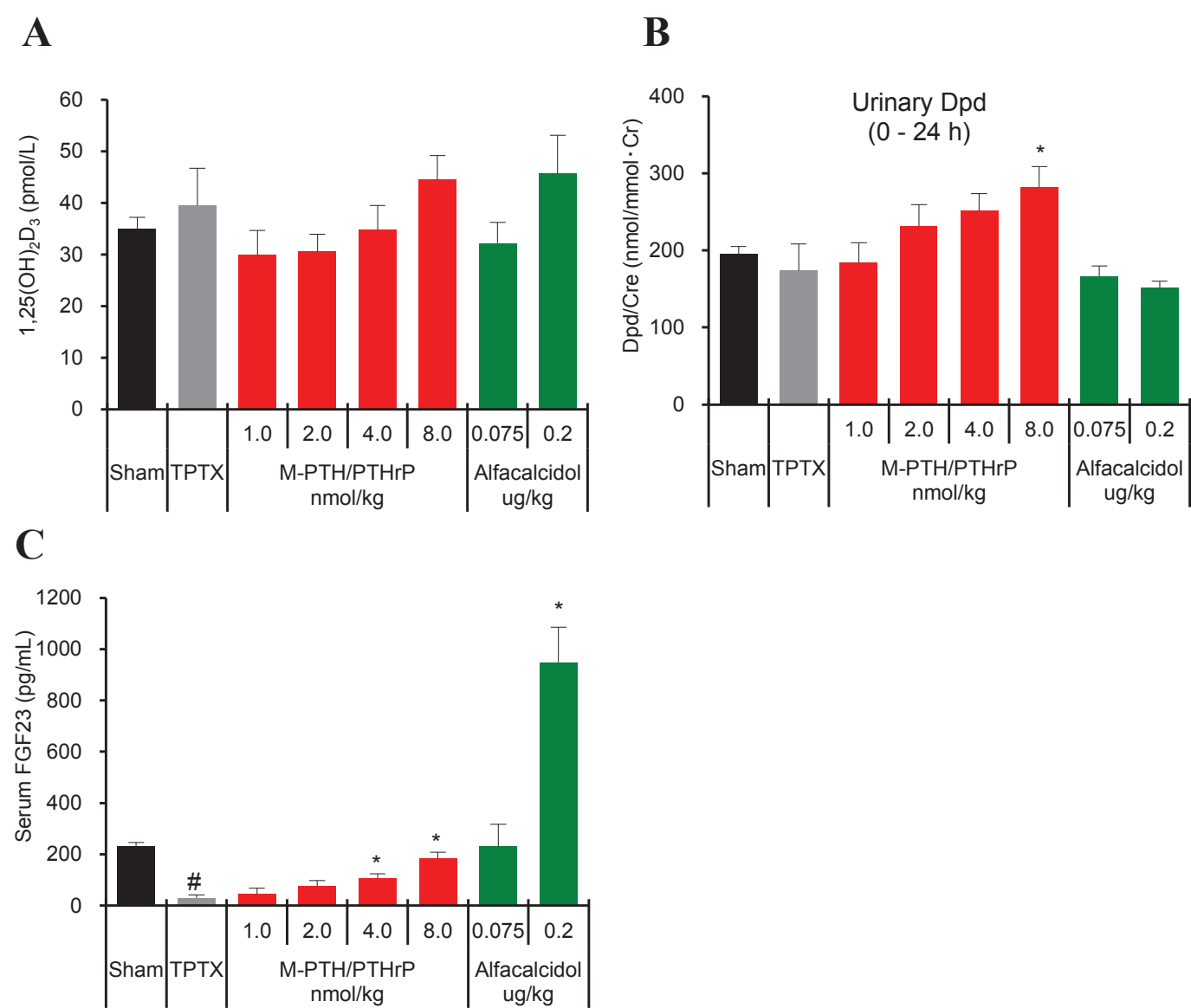

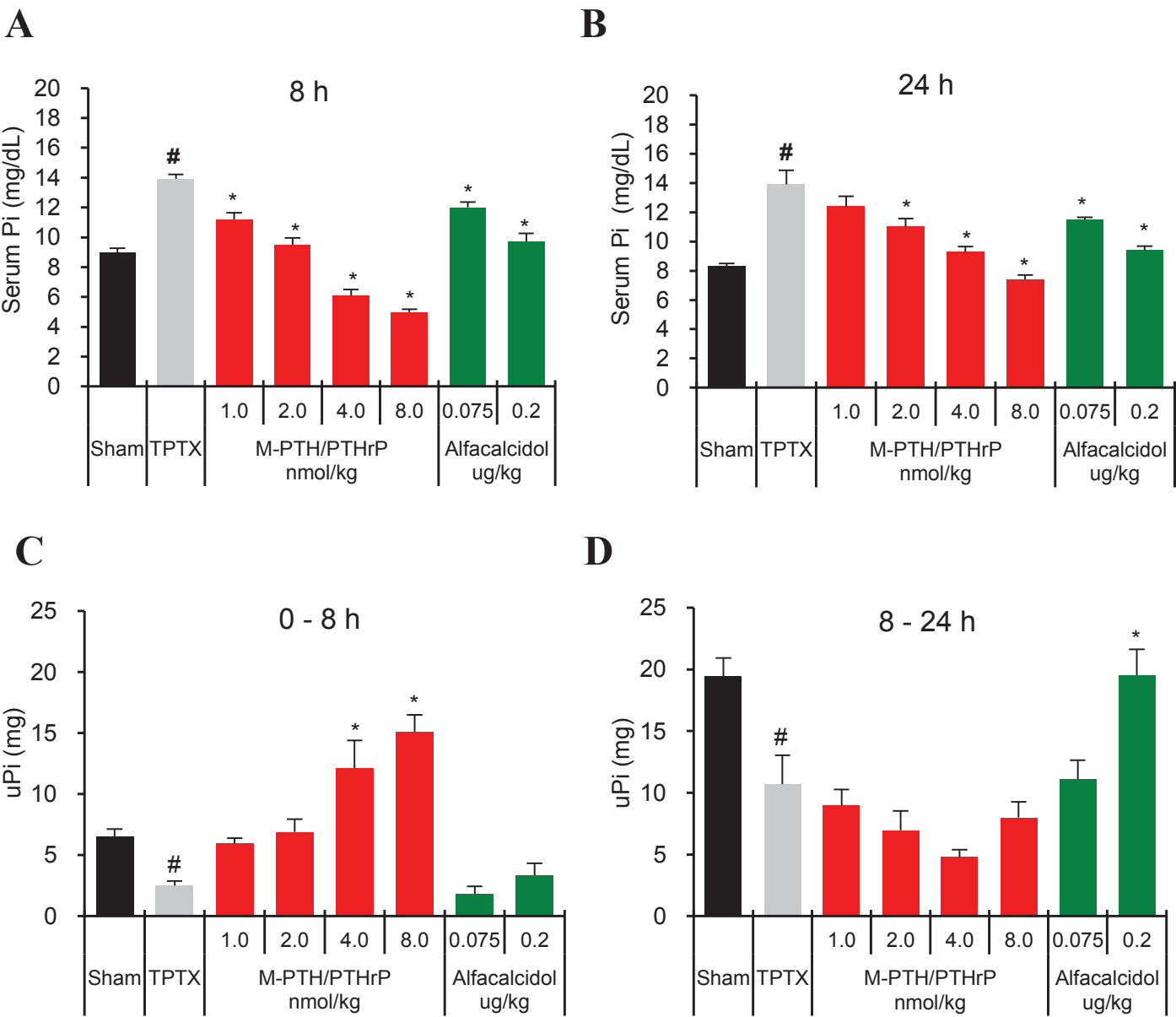

A

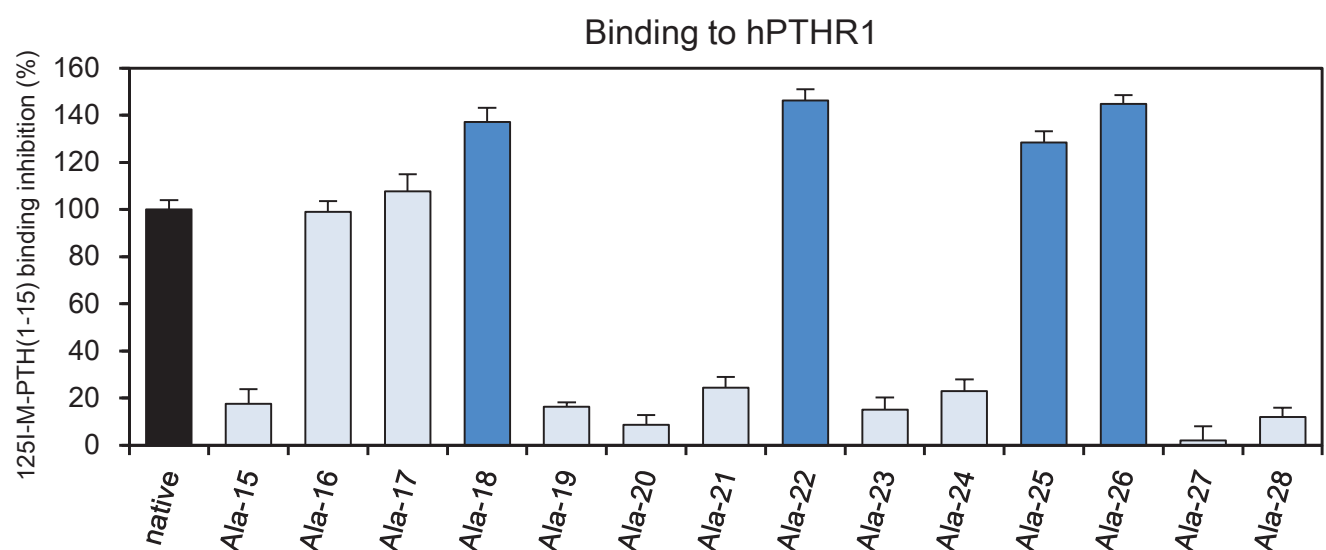

B

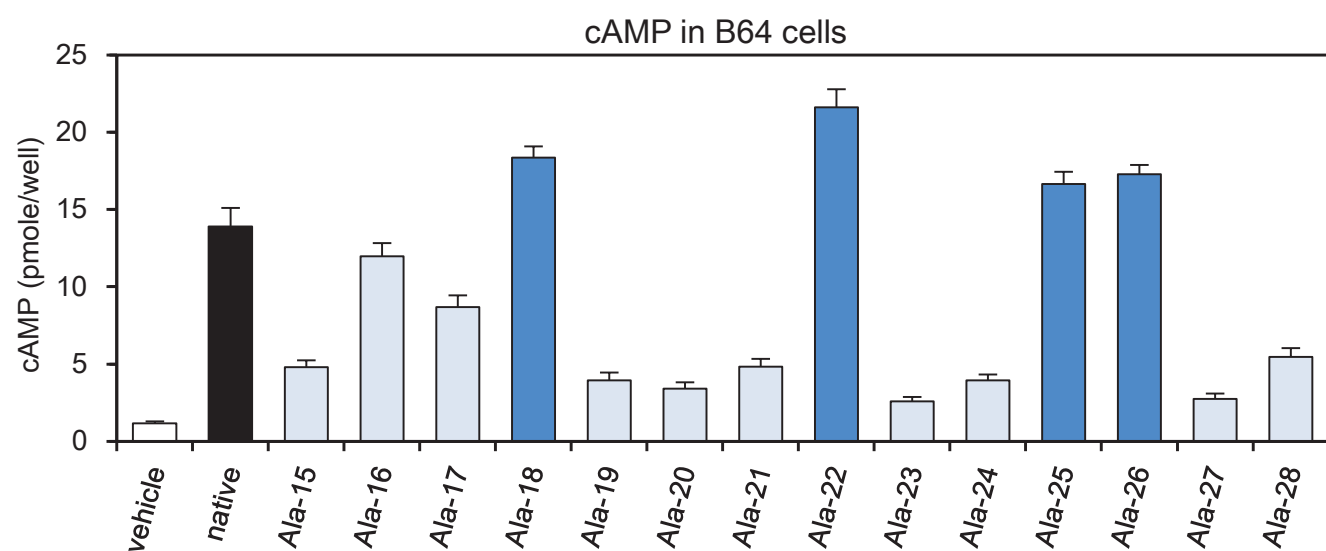

C

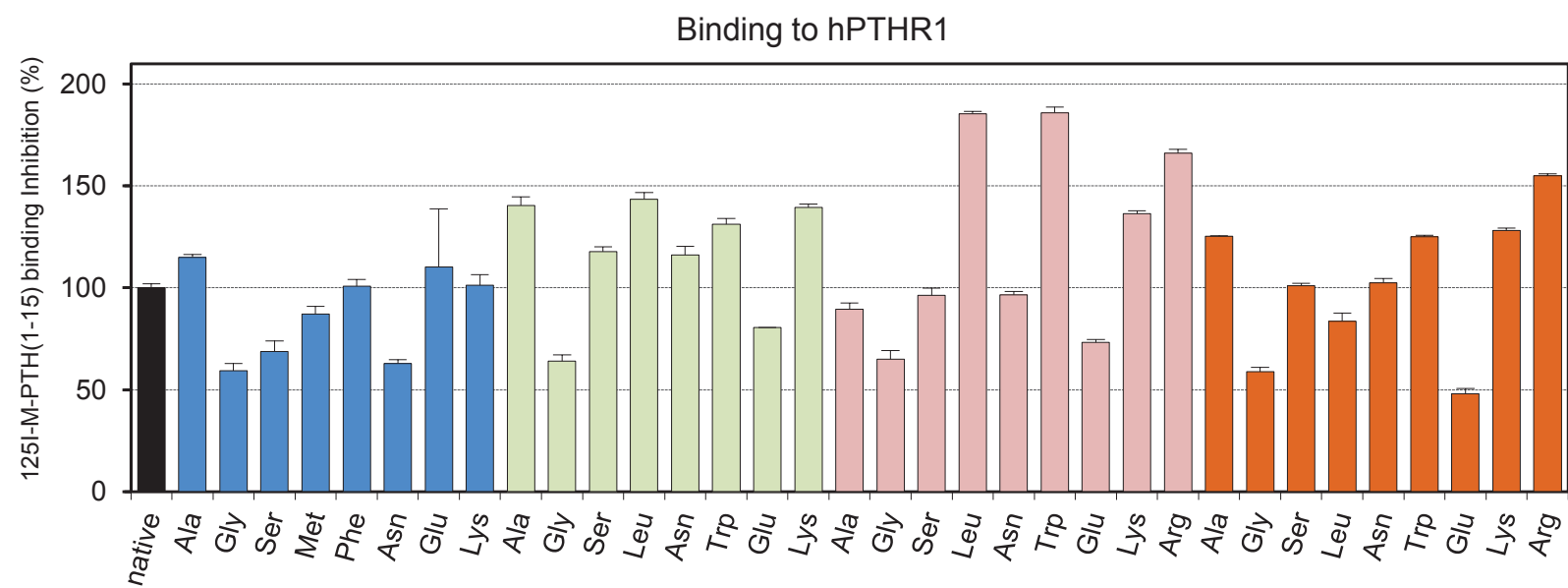

D

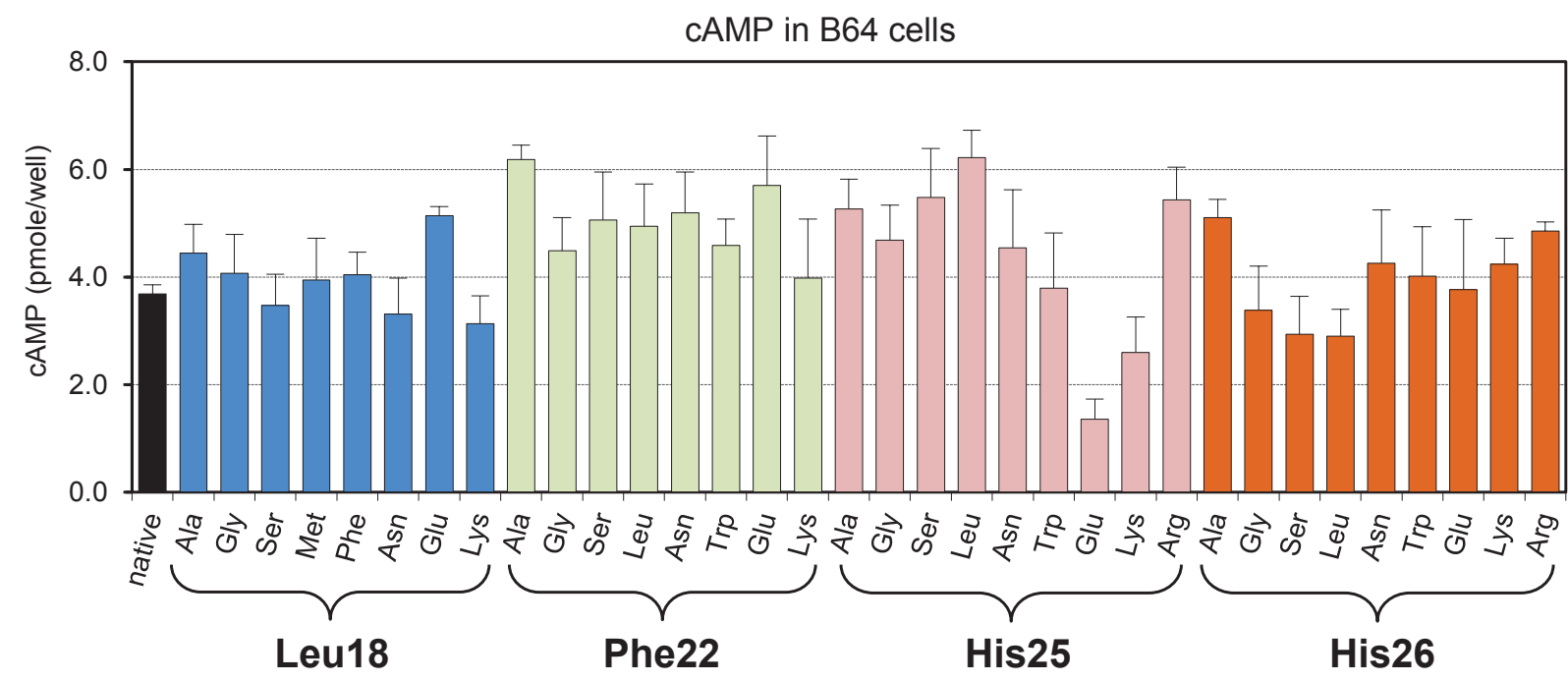

E

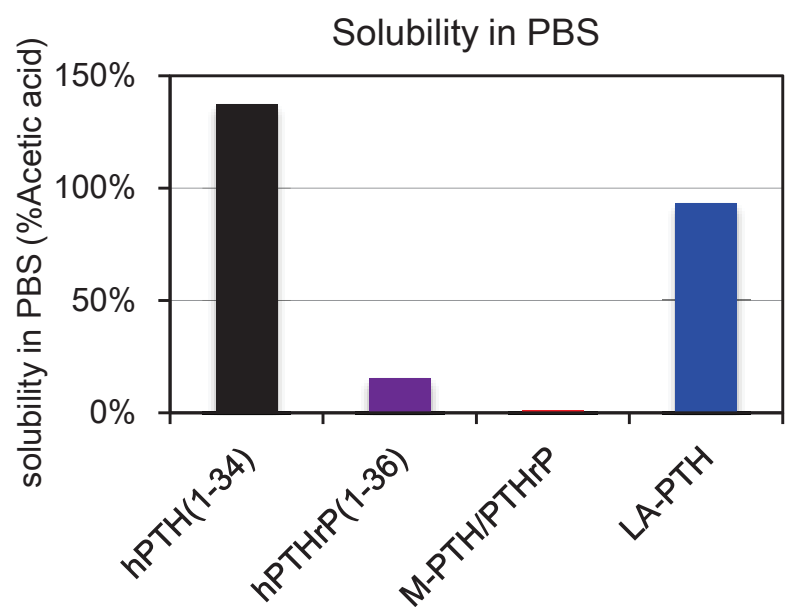

Supplement: Supplementary file 1 — Figure S1 PTH ligand‐induced cAMP‐signaling responses in GP‐2.3 cells Time course of the cAMP‐dependent luminescence response measured at 2‐minute intervals in the presence of each ligand at 10 nM (Ligand on‐phase) and B) Time course of the cAMP‐dependent luminescence response measured at 2‐minute intervals after washout of unbound ligand (Ligand wash‐out phase). GP‐2.3 cells preloaded with luciferin were treated with a concentration of PTH ligand at a concentration of 10 nM for 30 min, and cAMP‐dependent luminescence was measured at two‐minute intervals (ligand‐on phase). The cells were then removed from the plate reader, rinsed twice with media to remove unbound ligand, and then fresh media containing luciferin but lacking ligand was added and luminescence was assessed for additional 90 min (Ligand wash‐out phase). Data are means (± SEM) of four experiments, each performed in triplicate. Figure S2. In vivo calcemic actions of M‐PTH/PTHrP hybrid analogs in normal rats. The indicated M‐PTH/PTHrP hybrid analogs (sequences are shown in Figure 1) along with PTH(1–34) and PTHrP(1–36) controls, were administered at the indicated doses by i.v. injection, and at the indicated time points blood was collected from the tail vein and measured for ionized calcium (Ca2+). Panels A‐C represent three separate experiments. Data are means ± SEM; n = 6 or 4 (80 nmol/kg of M‐PTH(1‐11)/PTHrP(12–36), M‐PTH(1‐14)/PTHrP(12–36), M‐PTH(1‐17)/PTHrP(18–36) and 1.25 nmol/kg of M‐PTH(1–30)/PTHrP(31–36)). *p < 0.05 versus Vehicle. Figure S3. Effects of 11 days treatment (12 days study) of M‐PTH/PTHrP on serum 1,25(OH) 2 Vitamin‐D3, urinary deoxypyridinoline (Dpd) and serum FGF23 in TPTX rats. Serum and urine samples obtained from the TPTX and sham control rats of the two‐week daily‐administration experiment shown in Figure 4 were analyzed by ELISA for the indicated markers. A) serum 1,25(OH)2Vitamin‐D3. B) urinary deoxypyridinoline (DpD)/Cre. C) serum FGF23. Data are means ± SEM; n = 6 or 5 (2.0 [file JBM4-4-e10367-s001.pdf]
